# Strong Relation Between an EEG Functional Connectivity Measure and Postmenstrual Age: A New Potential Tool for Measuring Neonatal Brain Maturation

**DOI:** 10.3389/fnhum.2018.00286

**Published:** 2018-07-17

**Authors:** Laura Anna van de Pol, Charlotte van ’t Westende, Inge Zonnenberg, Esther Koedam, Ineke van Rossum, Willem de Haan, Marjan Steenweg, Elisabeth Catharina van Straaten, Cornelis Jan Stam

**Affiliations:** ^1^Department of Child Neurology, VU University Medical Center, Amsterdam, Netherlands; ^2^Department of Neonatology, VU University Medical Center, Amsterdam, Netherlands; ^3^Department of Neurology, VU University Medical Center, Amsterdam, Netherlands; ^4^Department of Clinical Neurophysiology, VU University Medical Center, Amsterdam, Netherlands

**Keywords:** electroencephalography, phase lag index, functional connectivity, maturation, neonate

## Abstract

Fetal and neonatal brain connectivity development is highly complex. Studies have shown that functional networks change dramatically during development. The purpose of the current study was to determine how the mean phase lag index (mPLI), a measure of functional connectivity (FC), assessed with electroencephalography (EEG), changes with postmenstrual age (PMA) during the early stages of brain development after birth. Neonates (*N* = 131) with PMA 27.6–45.3 weeks who underwent an EEG for a medical reason were retrospectively studied. For each recording, global FC was assessed by obtaining a whole-head average of all local PLI values (pairwise between sensor space EEG signals). Global FC results were consequently correlated with PMA values in seven frequency bands. Local results were obtained for the frequency band with the strongest global association. There was a strong negative correlation between mPLI and PMA in most frequency bands. The strongest association was found in the delta frequency band (*R* = −0.616, *p* < 0.001) which was therefore topographically explored; the strongest correlations were between pairs of electrodes with at least one electrode covering the central sulcus. Even in this heterogeneous group of neonates, global FC strongly reflects PMA. The decrease in PLI may reflect the process of segregation of specific brain regions with increasing PMA. This was mainly found in the central brain regions, in parallel with myelination of these areas during early development. In the future, there may be a role for PLI in detecting atypical FC maturation. Moreover, PLI could be used to develop biomarkers for brain maturation and expose segregation processes in the neonatal brain.

## Introduction

The development of the fetal and neonatal brain is highly complex. The brain follows an elaborate developmental trajectory, involving the formation of 10 billion cortical neurons, which migrate from their origin in the ventricular and the later-formed sub-ventricular zones to their final position and start to grow their own synaptic connections (van den Heuvel et al., 2015; Fernández et al., 2016). For example, at a postmenstrual age (PMA) of 20 weeks, a major structural change begins; the cortex expands greatly in surface area and becomes wrinkled and folded (Keeling, 2003; Bendersky et al., 2006; Rajagopalan et al., 2011). In addition, myelination is already seen in the white matter by 28 weeks, and between 34 and 46 weeks it becomes more prominently visible in the cortex bordering the central sulcus, extending in the posterior limb of the internal capsule (Sie et al., 1997; Counsell et al., 2002). These highly plastic changes make it a challenge to study the active neonatal brain, and to distinguish normal from abnormal development patterns.

A frequently used tool to study neonatal brain function is electroencephalography (EEG). EEG non-invasively records the electrical activity of the brain from the scalp. The signals that are recorded with the electrodes reflect synchronous neuronal activity of the cortex, in particular excitatory and inhibitory post-synaptic potentials of pyramidal cells in layer three and five related to synaptic currents. An advantage of this technique in neonates is that it can be applied at the bedside in the earliest postnatal phases at the neonatal intensive care unit. Visual assessment of the developing background pattern with increasing postmenstrual age is used in clinical practice. However, little is known about the development of functional brain networks in these early stages.

In the past decade, EEG has been increasingly used for “functional connectivity (FC)” analysis (Stam and van Straaten, 2012). FC can be defined as the temporal correlation between spatially distant neurophysiological events, and is a measure of functional communication between distant brain regions. EEG is a suitable tool for analyzing FC of the brain, because of the high temporal resolution between signals recorded from different brain areas.

Efficient functional communication between neuronal regions due to a gradual optimal organization of structural connections develops mostly after birth. Distant neural populations need to be connected by associational and callosal fibers, and the myelination of these fibers is only completed after the second decade of life. Previous studies have provided evidence that functional networks change more dramatically during development than structural (physical) networks. Especially during the first years of life, many studies point toward an increase in segregation (the neuronal processing carried out by separate groups of regions) and integration (the efficiency of global communication) of functional networks in the brain (see Cao et al., 2016 for a review). However, most of these studies are conducted in subjects with PMAs above 46 weeks, and little is known about how functional brain networks develop in the period between 24 and 46 weeks.

Although EEG is a very valuable clinical technique, it does have several methodological pitfalls. A common methodological problem with FC analysis is that time series recorded from different electrodes can reflect activity from common sources, yielding statistical interdependencies that are not representing FC of the brain. This is referred to by the problem of “volume conduction” (Guevara et al., 2005). The nonlinear FC measure “phase lag index” (PLI) diminishes the problem of volume conduction (Stam et al., 2007). The PLI is a measure for the asymmetry of the distribution of phase differences between two EEG signals; it reflects the consistency with which one signal is phase leading or lagging another signal. PLI discards phase differences that center around 0 mod π, reducing the effects of common sources by the principle that a non-zero phase lag cannot be explained by volume conduction. A number of studies have demonstrated that PLI can be successfully used as a measure for detecting differences between functional network organizations in a wide range of ages (Boersma et al., 2013; Engels et al., 2015; Smit et al., 2016; Yu et al., 2016; Sunwoo et al., 2017; van Lutterveld et al., 2017).

Recently, it has been shown that the PLI is sensitive to differences in brains of premature and full-term neonates at a PMA between 39 and 40 weeks in delta and beta frequency bands (González et al., 2011). In addition, networks built on PLI measures were strongly associated with gestational age (GA) in full-term healthy neonates with PMAs between 36 and 41 weeks (Tóth et al., 2017). Although these studies point towards a role for PLI as a measure of brain condition in neonates, little is known about PLI changes in the period before 36 weeks PMA, when the first brain networks start to develop, and changes in brain network organization are likely to occur on very short time scales.

The objective of this study is to investigate how the PLI develops over time during the very early stages of brain development in a diverse group of preterm and full-term born neonates who underwent an EEG in clinical practice. The relationship between PMA and PLI is retrospectively studied in a group of neonates with a PMA < 46 weeks during recording. We hypothesize that PLI reflects the strong plasticity of the development of the neonatal brain during the first weeks of life after birth.

## Materials and Methods

### Subjects

The group consisted of 131 subjects with PMA between 27–46 weeks, in whom an EEG had been recorded for a medical reason. Table 1 shows the patients characteristics. The most prevalent medical condition was hypoxic-ischemic encephalopathy due to perinatal asphyxia, and was defined as one or more of the following criteria: Apgar score at 5 min after birth ≤ 5; post-partum resuscitation; pH < 7.0 until 1 h post-partum; base excess < −16 mmol/ml until 1 h post-partum, lactate > 10.0 mmol/l until 1 h post partum and encephalopathy defined as a Thompson score ≥ 7 or a continuous function monitoring pattern showing flat-trace, burst-suppression or continuous low voltage.

**Table 1:** Patient characteristics.

	Subjects (*n* = 131)
Gender, male n (%)	72 (55)
Gestational age (weeks), median (range)	39.0 (24.0–42.4)
Birth weight*, median (range)	3130 (585–5500)
PMA during recording (weeks), median (range)	40.1 (27.6–45.3)
Age during recording (days), median (range)	5 (1–127)
Diagnosis, n (%)	
Asphyxia	42 (32.0)
Hemorrhage or infarction	19 (14.5)
Infection	8 (6.1)
Complication of prematurity	10 (7.6)
Cerebral malformation	8 (6.1)
Epileptic encephalopathy	4 (3.1)
Other	11 (8.4)
No diagnosis	29 (22.1)

*n = 6 missing values, PMA = postmenstrual age.

The research has been approved by the medical ethical committee of the VU University Medical Center (sub study of project: “*Brain network analysis in preterm infants using electro-encephalography—a pilot study*” Medical Ethical Committee protocol number: **2015.460** and NL-number NL54151.029.15). The medical ethical committee of the VUmc waived the necessity of an official consent form, since the procedures were part of a standard clinical practice.

### EEG Recording and Post-processing

EEG recordings from neonates with PMA 24–46 weeks, made between March 2012 and March 2017, were retrieved from the database of the Department of Clinical Neurophysiology of the VU University Medical Center, Amsterdam, using BrainRT (OSG digital equipment BrainRT; OSG b.v., Rumst, Belgium). In addition, to explore longitudinal PLI changes, subjects who had more than five EEG recordings over time before a PMA of 46 weeks, were selected from the database.

EEGs were recorded at the Department of Clinical Neurophysiology of the VU University Medical Center. 21 Ag/AgCl electrodes (Fp2, Fp1, F8, F7, F4, F3, A2, A1, T4, T3, C4, C3, T6, T5, P4, P3, O2, O1, Fz, Cz, Pz) were attached to the scalp at the positions of the International 10–20 system. Impedance was kept below 5 KOhm. Initial filters settings were a time constant of 0.6 s, resulting in a lower frequency cut-off of $\frac{1}{2\pi *time\;constant}$ = 0.265 Hz.

Signals were low-pass filtered at 100 Hz. The sample frequency was 500 Hz and EEG was digitized with 20 bit resolution. The average reference included all electrodes.

The EEG recordings were converted into ASCII-format, with the channels arranged according to the BrainWave software (version 0.9.152.4.1, available from http://home.kpn.nl/stam7883/brainwave.html) setting “Average” (see Supplementary Table S1). For subjects with more than one EEG recorded with PMA < 46 weeks, the first recording was used, and no further selection of EEGs was performed.

EEGs were downsampled to 250 Hz in BrainWave and the final epoch length consisted of 4096 samples (16.384 s). All channels and the first 50 epochs of each EEG recording were included for FC analysis and band-pass filtered into seven frequency bands: broad band: 0.1–45 Hz; delta: 0.5–4 Hz; theta: 4–8 Hz; lower alpha: 8–10 Hz; upper alpha: 10–13 Hz; beta: 13–30 Hz; gamma: 30–45 Hz.

For later data exploration, EEG quality was globally assessed by one of the authors (CvtW) based on epoch numbers; 1, 10, 20, 30, 40 and 50. During the visual assessment, EEG recordings were not filtered in one of the frequency bands. Unwanted non-brain signals such as electric fields generated by muscles or nearby equipment were indicated as significant artifacts when more than 30 percent of the epoch was affected by it. Quality of EEGs was scored as “high” when none or only one artifact was visually detected and “low” when two or more artifacts were visually detected. For clinical practice, EEGs were assessed by experienced clinical neurophysiologists and visually evaluated as “normal” or “abnormal.” EEGs were not categorized based on other criteria, such as sleep-states or pattern of EEG.

### EEG Data Analysis: The Phase Lag Index (PLI)

The FC measure PLI was computed between all EEG channel pairs. The PLI reflects how consistent the phase of one signal, recorded in one channel, is leading or lagging the phase of a signal recorded in another channel, and ranges between 0 (no consistent phase leading or lagging) and 1 (complete phase leading or lagging; see Supplementary Figure S1; Stam et al., 2007). The PLI is calculated using the following equation:

(1) $$PLI=\left|\langle sign\;\;\left[\sin\left(\Delta \varphi (t_{k})\right)\right]\rangle \right|$$

where Δ*φ*(*t_k_*) is the phase difference, calculated for time point *t*_k_ with *k* = 1 … N, dependent on the sampling frequency. *Sign* refers to the signum function, which translates a positive value for the phase difference into 1, a negative value for the phase difference into −1 and no phase difference into 0. 〈 〉 refers to the mean value and | | denotes absolute values. FC analysis was performed with BrainWave software (version 0.9.152.4.1, available from http://home.kpn.nl/stam7883/brainwave.html; see Supplementary Figure S1).

PLI values were further analyzed in two ways: (1) the mean PLI of all pairs of channels was calculated for each epoch, followed by calculation of the mean over the 50 epochs per subject (the resulting value is referred to as “mPLI” in this article), and (2) the PLI values for each separate pair of channels were averaged over the 50 epochs per subject, resulting in a matrix of 21 vs. 21 pair-wise PLI (pwPLI) values per subject. The global analyses (including all channels) were done separately for the seven frequency bands and the local analyses (including two channels) were performed in the frequency band with the strongest association between PMA and mPLI.

### Statistical Analysis

All statistical analyses were performed using Statistical Package for the Social Sciences (SPSS)^®^ version 19.0 for Windows. The level of significance was set at *p* < 0.005 for the global analyses and *p* < 1 × 10^−4^ for the local analyses to adjust for multiple comparisons. The relationship between PMA and mPLI was assessed by calculating the Pearson’s correlation coefficient for the frequency bands without outliers and by calculating Spearman’s ρ for the frequency bands with one or more outliers. Outliers were defined as those with z-scores > 3.29. In addition, a linear regression model, using the Enter method (Field, 2014), was constructed for the frequency band with the strongest association between mPLI and PMA. Furthermore, the influence of GA and birth weight was explored by calculating multiple regression models in the frequency band with the strongest correlation between mPLI and PMA. The influences of gender and asphyxia were assessed by means of a Mann-Whitney *U* test.

To evaluate the effect of EEG quality, Pearson’s correlation was calculated for the relationship between PMA and mPLI after selection of only high quality EEGs. Furthermore, to investigate the relationship between mPLI and PMA for EEG recordings that were clinically interpreted as normal, Pearson’s correlation was calculated.

To investigate the contribution to the total (broad band) power spectrum of the frequency band with the strongest association, power spectrum analysis was conducted using the Fast Fourier Transform option in Brainwave. The mean relative contribution was calculated over the 50 epochs per subject for the frequency band with the strongest association.

To extract information about the topography of the changes in mPLI with PMA, Pearson’s correlations were used, since topographical exploration was investigated in the frequency band with the strong linear correlation. Pearson’s correlations were calculated separately between all pwPLIs and PMA for the frequency band with the strongest association between mPLI and PMA. Scatterplots were checked for linearity and normally distributed data across the regression line.

Longitudinal changes in mPLI in relation to PMA within subjects were assessed by calculating Spearman’s ρ.

## Results

### Subjects

The final group consisted of 131 subjects with a median GA of 39.0 weeks (range: 24.00–42.43 weeks) and a median PMA at the moment of EEG recording of 40.14 weeks (range: 27.57–45.29 weeks). Table 1 shows a detailed description of the patient characteristics.

### Relationship Between mPLI and PMA

There was a strong negative correlation between PMA and mPLI in all frequency bands; correlations for all frequency bands are summarized in Table 2. The strongest association was found in the delta frequency band, with *R* = −0.616 and *p* < 0.001.

**Table 2:** Summary of correlations between PMA during recording and PLI.

	Correlation coefficient
Delta	−0.616^*#^
Theta	−0.391*
Lower alpha	−0.273*
Upper alpha	−0.270*
Beta	−0.290*
Gamma	−0.322*
Broadband	−0.350^*#^

PMA = postmenstrual age, PLI = phase lag index. *n* = 131. Values are Spearman’s r, except for values marked with # for which Pearson’s R was reported. *Significant at the *p* < 0.005 level.

**Figure 1:**
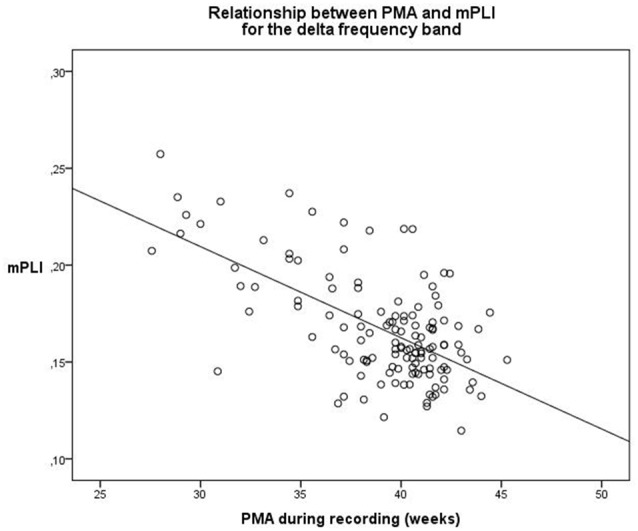
Relationship between PMA and mPLI in the delta frequency band. The linear relationship between PMA and PLI is shown for the regression model, with B0 = 0.344 and B1 = −0.005. Dots correspond to individual subjects (*N* = 131). PMA = postmenstrual age, mPLI = mean phase lag index.

A linear regression model was constructed for the delta frequency band. PMA explains a significant proportion of the variance in mPLI in the delta frequency band (*F*_(1,129)_ = 47.570, *p* < 0.001), with *R* = −0.616, *R*^2^ = 0.380. The regression coefficients for the intercept value and the slope value are significantly different from zero, with B0 = 0.344, *t*_(129)_ = 13.436, *p* < 0.001 and B1 = −0.005, *t*_(129)_ = −6.897, *p* < 0.001, respectively. The scatterplot is shown in Figure 1.

Multiple regression analysis was used to test if GA significantly predicted variance in mPLI in addition to PMA in the delta frequency band. The results of the regression indicated that GA did not significantly predict more of the variance in mPLI (R^2^change = 0.011, *F*_(1,128)_ = 2.257, *p* = 0.135).

Adding birth weight to the model with PMA and to the model with PMA and GA did not significantly predict more of the variance in mPLI (R^2^change = 0.026, *F*_(1,122)_ = 5.241, *p* = 0.024, and R^2^change = 0.014, *F*_(1,122)_ = 2.933, *p* = 0.089, respectively).

When comparing males vs. females, and asphyxia patients vs. patients without asphyxia, no differences in mPLI values in the delta frequency band were found (*U* = 2280.5, *z* = 0.724, *p* = 0.724 and *U* = 3169.0, *z* = 0.096, *p* = 0.924, respectively).

**Figure 2:**
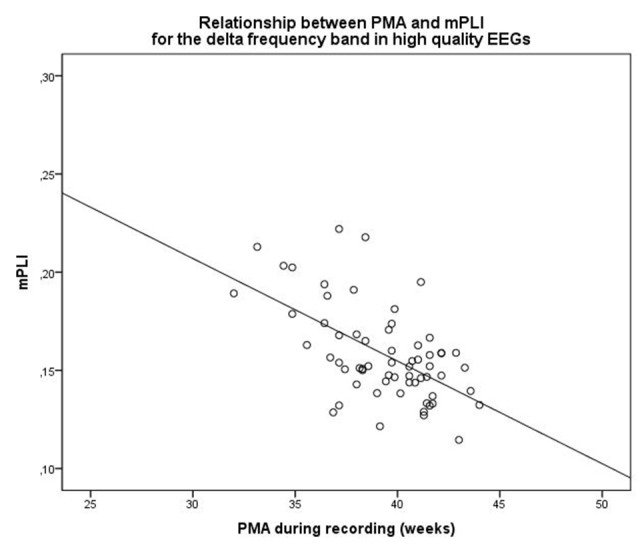
Scatterplot of PMA and mPLI in the delta frequency band, in a subgroup of subjects with EEGs assessed as high quality. Dots correspond to individual subjects (*N* = 63). PMA = postmenstrual age, mPLI = mean phase lag index.

When selecting only high-quality EEGs, the group consisted of 63 subjects with a median PMA of 39.71 weeks (range: 32.0–44.0 weeks). There was a strong correlation between mPLI and PMA, with *R* = −0.571, *p* < 0.001 in the delta frequency band. The scatterplot is shown in Figure 2.

**Figure 3:**
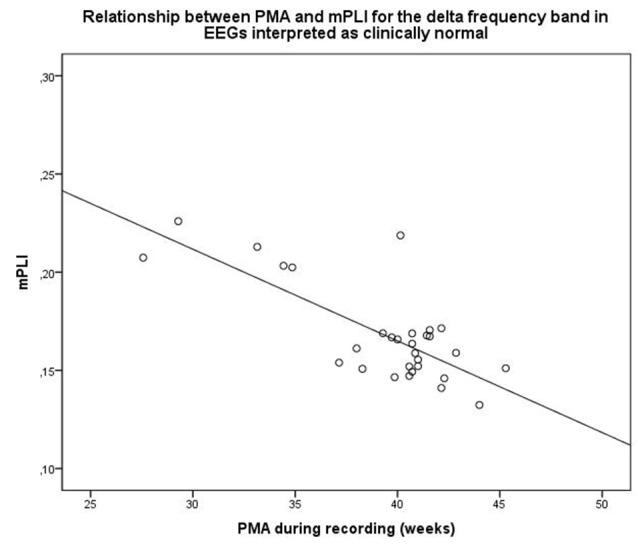
Scatterplot of PMA and mPLI in the delta frequency band in a subgroup with EEG clinically interpreted as normal. Dots correspond to individual subjects (*N* = 30). PMA = postmenstrual age, mPLI = mean phase lag index.

For EEGs clinically interpreted as normal (*N* = 30, mean PMA: 39.4 weeks, range PMA: 27.0–46.0 weeks), a strong relationship between mPLI and PMA with *R* = −0.757, *p* < 0.001 was found. The scatterplot is shown in Figure 3.

A power spectrum analysis was conducted to assess the percentage of delta in the power spectrum. One-hundred and twenty-four subjects (95%) had more than 60 percent of delta power in their EEG recording. Supplementary Figure S2 shows the power spectra of the individuals and the average power spectrum of the group.

### The Topography of the Changes in PLI With PMA

**Figure 4:**
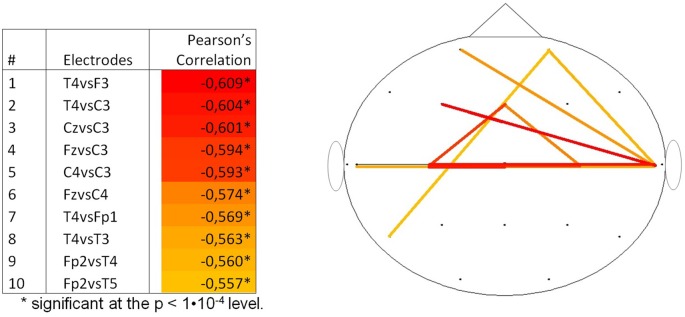
Top 10 correlations per couple of channels between pwPLI and PMA for the delta frequency band (*N* = 131). PMA = postmenstrual age, pwPLI = pair wise phase lag index.

By calculating the Pearson’s correlation between the pwPLI values and PMA, information on the topography of the changes in FC was extracted for the delta frequency band. Supplementary Table S2 shows all the Pearson’s correlations between PMA and pwPLI values. Figure 4 shows the 10 highest negative correlations on the scalp, with colors ranging from yellow (the 10th highest negative correlation) to red (the highest negative correlation). The highest negative correlations (with PMA) were the functional connectivity between electrodes T4 and F3, T4 and C3 and Cz and C3 (*R* = −0.609, *p* < 0.001, *R* = −0.604, *p* < 0.001 and *R* = −0.601, *p* < 0.001, respectively).

### Longitudinal Assessment of mPLI

In two subjects more than five EEGs were recorded. The first subject, with a diagnosis of a severe hemi-lissencephaly and intractable epilepsy, had eight EEG recordings, made between 41.1 and 52.3 weeks PMA. A trend towards a positive correlation between mPLI and PMA in the delta band was found, however this was not significant (*ρ* = 0.667, *p* = 0.071).

**Figure 5:**
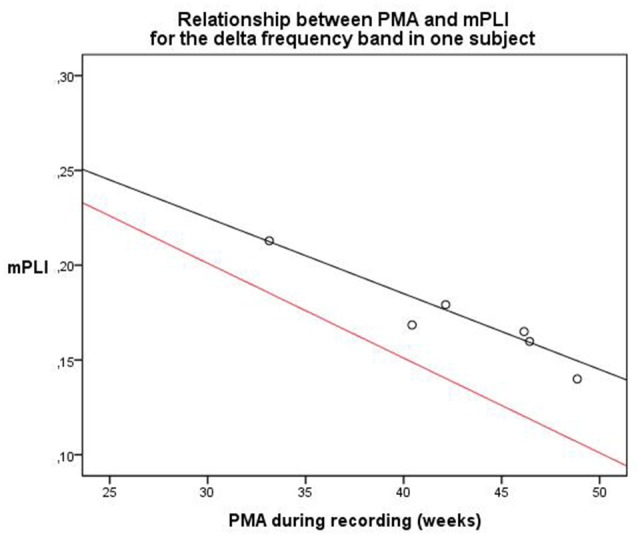
Relationship between PMA and mPLI in the delta frequency band in one subject at six different time points. The equation line of the resulting linear regression model is shown in black. The equation line of the regression model of the relation between PLI and PMA of the total group, based on the cross-sectional data, is shown in red as a reference. PMA = postmenstrual age, mPLI = mean phase lag index.

The second subject, with a diagnosis of cortical dysplasia causing epilepsy, had six EEG recordings, made between 33.1 and 48.9 weeks PMA. There was a significant negative correlation (*ρ* = −0.943, *p* = 0.005). A linear regression model was constructed; PMA explains a significant proportion of the variance in mPLI in the delta frequency band (*F*_(1,4)_ = 35.896, *p* = 0.004), with *R* = −0.949, R^2^ = 0.900. The regression coefficients for the intercept value and the slope value are significantly different from zero, with B0 = 0.345, *t*_(4)_ = 11.773, *p* < 0.001 and B1 = −0.004, *t*_(4)_ = −5.991, *p* = 0.004, respectively. The scatterplot is given in Figure 5, including a reference line from the linear regression model of the total group in the delta frequency band.

## Discussion

To date, there are few studies that have used FC measures to investigate how the neonatal brain develops. The purpose of the current study was to determine how the mPLI changes with age during the very early stages of brain development after birth. Our main finding was the strong decrease in mPLI with maturation from PMA 27–46 weeks in most frequency bands, even in this diverse group of subjects. The strongest correlation between mPLI and PMA was found in the delta frequency band, which also had the highest power in 95% of the subjects. Other functional connectivity studies in neonates have found significant results in the delta frequency band as well (González et al., 2011; Tokariev et al., 2016; Tóth et al., 2017), in line with our findings that the delta frequency band is (one of) the most interesting frequency bands to study in the neonatal brain.

Several graphoelements that partly consist of delta activity appear and disappear in the EEG during the period from PMA 27–46 weeks. For example, central delta brushes are usually seen from PMA 28–38 weeks, while anterior delta runs are usually seen from PMA 31–44 weeks (Tsuchida et al., 2013; Whitehead et al., 2017). This could influence functional connectivity outcomes that are based on the PLI measure. In addition, it is thought that various temporal frequencies in the EEG, including delta frequencies, reflect different neural sources (Pedley, 1997; Buzsaki and Draguhn, 2004). However, it remains unclear what neuronal mechanisms underlie the significant contribution of delta activity to the neonatal EEG, as well as in graphoelements as in functional connectivity.

The decrease of the FC measure mPLI with age may reflect an increase of segregation in the neonatal brain, with less centralized connections. This was also found in an EEG based network study by Meijer et al. (2014) demonstrating a decrease in coherence between the hemispheres with maturation in neonates. They speculated that this reflected the increasing complexity of the neuronal network with maturation. In line with this finding our study showed that six out of the ten strongest correlations between PLI and PMA was between interhemispherical pairs of electrodes indicating differentiation between the hemispheres. Another study, in which correlations between frequency-specific amplitudes were calculated, found frontal modules (subgroups of cortical regions) in full-term neonates (GA: 37.6–43.5 weeks), whereas these were not present in preterm neonates (GA: 28.3–33.8 weeks), again supporting the idea of segregation processes occurring in early stages (Omidvarnia et al., 2014).

Further topographical exploration of the negative correlation between mPLI and PMA in the delta frequency band revealed that the strongest correlations were between pairs of electrodes that were both on the central sulcus, or between pairs of electrodes of which one was on the central sulcus and one was on a frontal region. We speculate that this pattern might reflect the current concept of myelination of the neonatal brain, with brain maturation starting in the central area and proceeding towards the parieto-occipital cortex and corticospinal tracts of the pre- and postcentral gyri as the regions with the strongest increase in myelination during this period (Sie et al., 1997; Ruoss et al., 2001; Counsell et al., 2002), as a substrate for the increasing segregation of brain functions.

In contrast to our findings, Koolen et al. (2016) report an increase in synchronization of early cortical activity across the neocortex in premature infants with PMAs between 30 and 44 weeks. They used the activation synchrony index (ASI) which statistically quantifies the temporal coincidence of bursts in the cortical activity. A possible explanation for this discrepancy is that results derived from the ASI cannot be compared to results derived from the PLI, since the ASI focuses on bursts only, and the PLI on the overall EEG. Eiselt et al. (2001) have shown that burst periods provide higher coherence values than interburst periods. In addition, the lowest values of coherence were observed just before the burst started and during the burst, maximal coherence was reached at different times in different frequency bands. This suggests that segmentation of continuous EEG would affect the outcome of functional connectivity, since bursts could be cut into half. However, since the cuts are arbitrary and 50 epochs are used to calculate the mPLI in this study, this influence is minimized.

Several questions remain unanswered at present. To develop a full picture of the integration and segregation processes in the neonatal brain, additional studies are needed that take into account different FC measures, such as the Newman’s modularity and measures derived from minimum spanning trees for example (Newman, 2006; Stam et al., 2014).

Several studies have shown that EEG can be used as a sensitive and objective evaluation of the severity of brain injury related to asphyxia (Mariani et al., 2008; Nash et al., 2011; Briatore et al., 2013). Therefore, it was an unanticipated finding that asphyxia had no significant effect on functional connectivity outcome in our study. A possible explanation for this might be that the changes in EEG patterns of neonates with asphyxia are related to other aspects of the EEG, such as background amplitude, and that functional connectivity in the delta frequency band stays unaffected. Furthermore, it is difficult to draw general conclusions from the results of the longitudinal analyses, since only two subjects were investigated.

Studying FC in neonates with the use of EEG recordings is technically challenging, due to highly plastic changes in the brain during this period. Researchers use a variety of selection criteria for analyzing FC, including selections based on sleep-states and EEG quality (Grieve et al., 2008; González et al., 2011; Omidvarnia et al., 2014; Koolen et al., 2016; Tokariev et al., 2016; Tóth et al., 2017). Background patterns change dramatically with PMA in the neonatal period from discontinuous to more continuous patterns. EEG phenomena recorded during the early periods of discontinuity are thought to result from input generated spontaneously at the sensory periphery rather than by cortico-cortical connections, which makes it difficult to understand what changes in FC measures in the neonatal period actually indicate (Colonnese and Khazipov, 2012). In our explorative study no selection based on sleep states was made. Nevertheless, a strong correlation between mPLI and PMA was found. It remains unclear to what extent the age related changes in background pattern or sleep stages have contributed to the observed decrease in mPLI with age.

The influence of common sources on the ability of the PLI to detect real changes in synchronization is studied in different montages by Stam et al. (2007). They showed that PLI values were hardly influenced by different montages during pre-seizure epochs, and intermediately influenced by average referencing during seizure epochs. The PLI was less influenced by different montages (average, source, mastoids, Cz, bipolar) than the phase coherence and the imaginary part of coherence (Stam et al., 2007). Peraza et al. (2012) investigated the effects of volume conduction on PLI networks. They concluded that PLI networks based on independent sources in a volume conduction environment were different from random networks, providing evidence that volume conduction affects PLI inference in general. However, they investigated the performance of PLI networks and not PLI as a functional connectivity measure (Peraza et al., 2012).

In this study, we investigated the influence of artifacts by comparing outcomes between the total group without any kind of selection and the group with only high quality EEGs, which did not influence our results. This observation could be used to argue against arbitrary selection of EEG epochs when using the PLI, as long as a sufficiently large number of epochs is included in the analysis. However, there still is abundant room for further progress in determining the best ways to analyze FC in neonatal EEG recordings.

In addition to the strengths of our study (the large number of neonates included in this study and the very young PMA at the time of EEG recording) there are some drawbacks of this study. This retrospective study was conducted in a heterogeneous group of neonates, who had an indication for an EEG, with a wide range of clinical characteristics and administered medication, possibly influencing the EEG. On the other hand it is interesting that even despite this heterogeneity such a strong negative correlation between mPLI and PMA could be demonstrated. Interestingly, the strength of the negative correlation between mPLI and PMA increased when we repeated the analyses in a subgroup with EEGs that were clinically interpreted as normal, indicating that this relation might even be stronger when assessed in a healthy subgroup. Additionally, other possible influences, such as changes in head circumference, on the FC analysis were not completely unraveled in this study. A prospective EEG study including healthy and preterm neonates with a relatively uncomplicated clinical course is currently carried out to replicate this data.

This study contributes to the fundamental understanding of FC dynamics in the neonatal brain. We conclude that the PLI could serve as an objective measure for cerebral maturation that exposes segregation processes of neural circuits, resulting in better understanding of how the brain matures. Because of the high sensitivity of mPLI for PMA, PLI could possibly be used for detecting atypical FC maturation of the brain, on group or even subject-specific level. Moreover, the PLI may play a role in developing markers for brain maturation for future treatment strategies. Subjects at risk could be followed in time which may contribute to earlier detection of pathology and adequate long term prognosis of psychomotor development.

## Author Contributions

LAP: concept and design of the project, execution, analysis and interpretation of data, drafting and writing of the research report. CW: execution, analysis and interpretation of data, drafting and writing of the research report. IZ: concept and design of the project, revising of the research report. EK and IR: data analysis, revising of the research report. WH: interpretation of data, revising of the research report. MS and ECS: revising of the research report. CJS: concept and design of the project, interpretation of data, drafting and revising the research report. All authors approved the final article.

## Conflict of Interest Statement

The authors declare that the research was conducted in the absence of any commercial or financial relationships that could be construed as a potential conflict of interest.

## Abbreviations

FC: Functional connectivity
mPLI: mean Phase lag index
PMA: post-menstrual age
pwPLI: pair-wise PLI.
