# Supplementary material for: Strong Relation Between an EEG Functional Connectivity Measure and Postmenstrual Age: A New Potential Tool for Measuring Neonatal Brain Maturation
Source: Front Hum Neurosci. 2018 Jul 17;12:286. doi: 10.3389/fnhum.2018.00286 (PMC6056611; doi:10.3389/fnhum.2018.00286)
Supplement: Supplementary file 1 [file Data_Sheet_1.docx]

Supplementary Material

**Strong relation between an EEG functional connectivity measure and postmenstrual age: a new potential tool for measuring neonatal brain maturation**

Van de Pol LA^*^, van ’t Westende C, Zonnenberg IA

*** Correspondence:** LA van de Pol, l.vandepol@vumc.nl

**1 Supplementary Figures and Tables**

Supplementary Table 1 Channel arrangement for conversion of EEG recordings in BrainRT into ASCII-format.

| Channel | Electrode |
| --- | --- |
| 1 | Fp2-Avg (500 Hz) |
| 2 | Fp1-Avg (500 Hz) |
| 3 | F8-Avg (500 Hz) |
| 4 | F7-Avg (500 Hz) |
| 5 | F4-Avg (500 Hz) |
| 6 | F3-Avg (500 Hz) |
| 7 | A2-Avg (500 Hz) |
| 8 | A1-Avg (500 Hz) |
| 9 | T4-Avg (500 Hz) |
| 10 | T3-Avg (500 Hz) |
| 11 | C4-Avg (500 Hz) |
| 12 | C3-Avg (500 Hz) |
| 13 | T6-Avg (500 Hz) |
| 14 | T5-Avg (500 Hz) |
| 15 | P4-Avg (500 Hz) |
| 16 | P3-Avg (500 Hz) |
| 17 | O2-Avg (500 Hz) |
| 18 | O1-Avg (500 Hz) |
| 19 | Fz-Avg (500 Hz) |
| 20 | Cz-Avg (500 Hz) |
| 21 | Pz-Avg (500 Hz) |
| 22 | ECG-Avg (500Hz) |


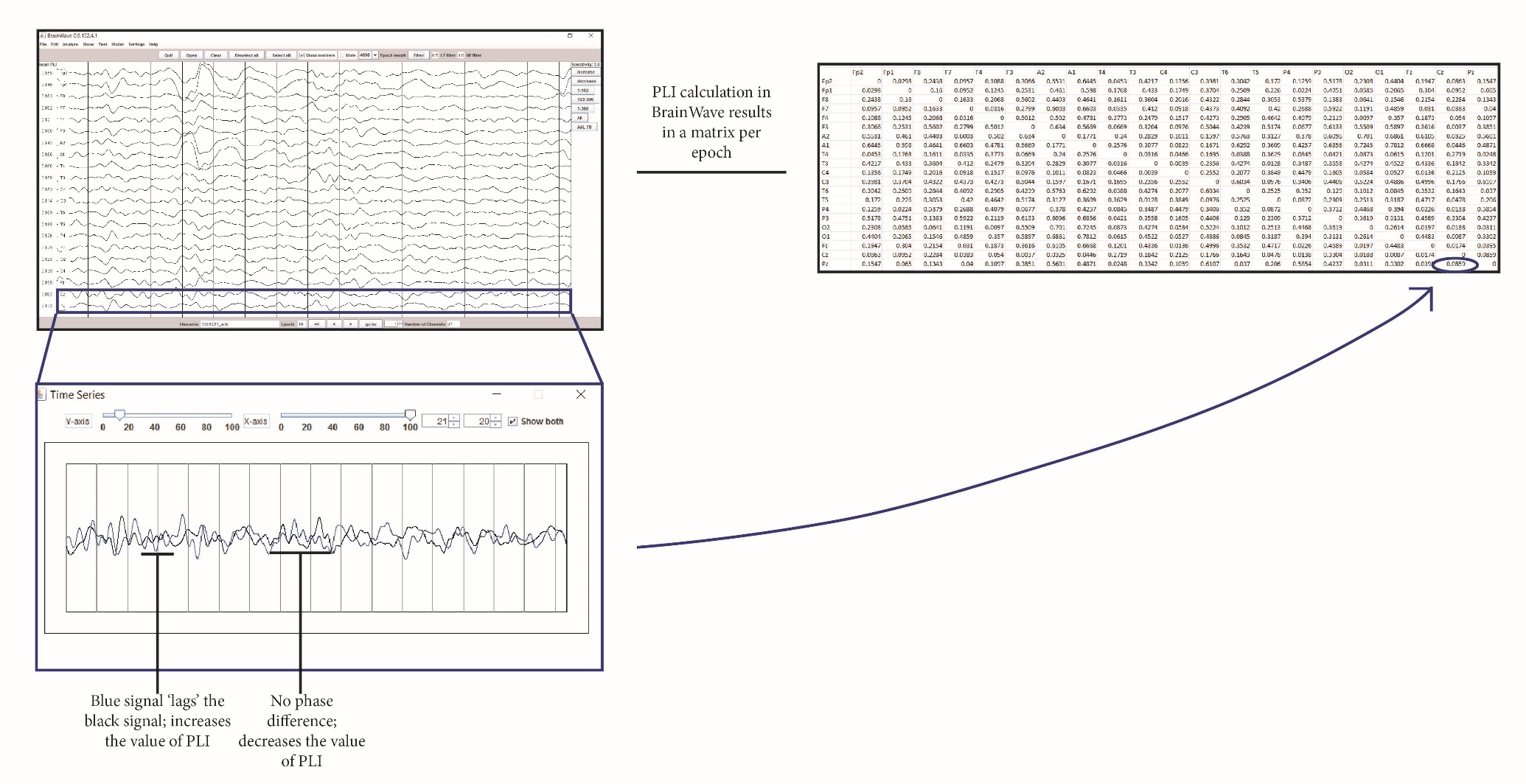


Supplementary Figure 1 Example of calculation of the PLI using the BrainWave software (version 0.9.152.4.1, available from <http://home.kpn.nl/stam7883/brainwave.html>). The analysis of two channels from one epoch is pointed out. PLI = phase lag index.

A)

B)

Supplementary Figure 2 Power spectrum analysis, with a) the power contribution per frequency band averaged over 50 epochs per subject (lines correspond to individual subjects) and b) the average spectrum for the group with corresponding standard errors (N = 131).

Supplementary Table 2 Pearson’s correlations between PMA and pwPLI values for the upper delta frequency band (N = 131). The colors range from white (no correlation) to red (highest correlations). Red font color indicates that the correlation was not significant at the p < 1•10^-4^ level. PMA = postmenstrual age, pwPLI = pair-wise phase lag index.

|  | Fp2 | Fp1 | F8 | F7 | F4 | F3 | A2 | A1 | T4 | T3 | C4 | C3 | T6 | T5 | P4 | P3 | O2 | O1 | Fz | Cz | Pz |
| --- | --- | --- | --- | --- | --- | --- | --- | --- | --- | --- | --- | --- | --- | --- | --- | --- | --- | --- | --- | --- | --- |
| Fp2 |  | -0.364 | -0.302 | -0.434 | -0.446 | -0.435 | -0.379 | -0.484 | -0.56 | -0.529 | -0.439 | -0.47 | -0.475 | -0.557 | -0.437 | -0.443 | -0.351 | -0.405 | -0.458 | -0.425 | -0.41 |
| Fp1 | -0.364 |  | -0.359 | -0.396 | -0.444 | -0.467 | -0.343 | -0.464 | -0.569 | -0.488 | -0.491 | -0.411 | -0.517 | -0.554 | -0.357 | -0.477 | -0.293 | -0.501 | -0.459 | -0.456 | -0.311 |
| F8 | -0.302 | -0.359 |  | -0.465 | -0.304 | -0.411 | -0.268 | -0.277 | -0.397 | -0.425 | -0.46 | -0.507 | -0.494 | -0.348 | -0.303 | -0.328 | -0.334 | -0.313 | -0.418 | -0.527 | -0.338 |
| F7 | -0.434 | -0.396 | -0.465 |  | -0.393 | -0.376 | -0.251 | -0.341 | -0.481 | -0.371 | -0.515 | -0.462 | -0.503 | -0.513 | -0.316 | -0.341 | -0.241 | -0.413 | -0.435 | -0.359 | -0.337 |
| F4 | -0.446 | -0.444 | -0.304 | -0.393 |  | -0.518 | -0.338 | -0.336 | -0.48 | -0.411 | -0.393 | -0.401 | -0.448 | -0.398 | -0.235 | -0.365 | -0.406 | -0.476 | -0.356 | -0.301 | -0.296 |
| F3 | -0.435 | -0.467 | -0.411 | -0.376 | -0.518 |  | -0.433 | -0.47 | -0.609 | -0.485 | -0.516 | -0.493 | -0.336 | -0.371 | -0.348 | -0.41 | -0.382 | -0.419 | -0.297 | -0.458 | -0.237 |
| A2 | -0.379 | -0.343 | -0.268 | -0.251 | -0.338 | -0.433 |  | -0.232 | -0.218 | -0.399 | -0.422 | -0.392 | -0.313 | -0.322 | -0.275 | -0.339 | -0.301 | -0.347 | -0.36 | -0.327 | -0.176 |
| A1 | -0.484 | -0.464 | -0.277 | -0.341 | -0.336 | -0.47 | -0.232 |  | -0.443 | -0.377 | -0.469 | -0.464 | -0.421 | -0.329 | -0.317 | -0.323 | -0.227 | -0.277 | -0.418 | -0.318 | -0.347 |
| T4 | -0.56 | -0.569 | -0.397 | -0.481 | -0.48 | -0.609 | -0.218 | -0.443 |  | -0.563 | -0.472 | -0.604 | -0.355 | -0.47 | -0.466 | -0.553 | -0.535 | -0.471 | -0.531 | -0.399 | -0.48 |
| T3 | -0.529 | -0.488 | -0.425 | -0.371 | -0.411 | -0.485 | -0.399 | -0.377 | -0.563 |  | -0.519 | -0.421 | -0.431 | -0.416 | -0.452 | -0.427 | -0.418 | -0.496 | -0.449 | -0.367 | -0.396 |
| C4 | -0.439 | -0.491 | -0.46 | -0.515 | -0.393 | -0.516 | -0.422 | -0.469 | -0.472 | -0.519 |  | -0.593 | -0.457 | -0.483 | -0.43 | -0.469 | -0.387 | -0.45 | -0.574 | -0.292 | -0.42 |
| C3 | -0.47 | -0.411 | -0.507 | -0.462 | -0.401 | -0.493 | -0.392 | -0.464 | -0.604 | -0.421 | -0.593 |  | -0.491 | -0.386 | -0.487 | -0.358 | -0.383 | -0.401 | -0.594 | -0.601 | -0.372 |
| T6 | -0.475 | -0.517 | -0.494 | -0.503 | -0.448 | -0.336 | -0.313 | -0.421 | -0.355 | -0.431 | -0.457 | -0.491 |  | -0.37 | -0.476 | -0.403 | -0.351 | -0.37 | -0.491 | -0.371 | -0.378 |
| T5 | -0.557 | -0.554 | -0.348 | -0.513 | -0.398 | -0.371 | -0.322 | -0.329 | -0.47 | -0.416 | -0.483 | -0.386 | -0.37 |  | -0.355 | -0.311 | -0.325 | -0.355 | -0.357 | -0.404 | -0.305 |
| P4 | -0.437 | -0.357 | -0.303 | -0.316 | -0.235 | -0.348 | -0.275 | -0.317 | -0.466 | -0.452 | -0.43 | -0.487 | -0.476 | -0.355 |  | -0.325 | -0.394 | -0.504 | -0.362 | -0.364 | -0.339 |
| P3 | -0.449 | -0.477 | -0.328 | -0.341 | -0.365 | -0.41 | -0.339 | -0.323 | -0.553 | -0.427 | -0.469 | -0.358 | -0.403 | -0.311 | -0.325 |  | -0.502 | -0.462 | -0.444 | -0.391 | -0.313 |
| O2 | -0.351 | -0.293 | -0.334 | -0.241 | -0.406 | -0.382 | -0.301 | -0.227 | -0.535 | -0.418 | -0.387 | -0.383 | -0.351 | -0.325 | -0.394 | -0.502 |  | -0.415 | -0.507 | -0.366 | -0.347 |
| O1 | -0.405 | -0.501 | -0.313 | -0.413 | -0.476 | -0.419 | -0.347 | -0.277 | -0.471 | -0.496 | -0.45 | -0.401 | -0.37 | -0.355 | -0.504 | -0.462 | -0.415 |  | -0.545 | -0.448 | -0.428 |
| Fz | -0.458 | -0.459 | -0.418 | -0.435 | -0.356 | -0.297 | -0.36 | -0.418 | -0.531 | -0.449 | -0.574 | -0.594 | -0.491 | -0.357 | -0.362 | -0.444 | -0.507 | -0.545 |  | -0.447 | -0.375 |
| Cz | -0.425 | -0.456 | -0.527 | -0.359 | -0.301 | -0.458 | -0.327 | -0.318 | -0.399 | -0.367 | -0.292 | -0.601 | -0.371 | -0.404 | -0.364 | -0.391 | -0.366 | -0.448 | -0.447 |  | -0.257 |
| Pz | -0.41 | -0.311 | -0.338 | -0.337 | -0.296 | -0.237 | -0.176 | -0.347 | -0.48 | -0.396 | -0.42 | -0.372 | -0.378 | -0.305 | -0.339 | -0.313 | -0.347 | -0.428 | -0.375 | -0.257 |  |
